# Combining Path Integration and Remembered Landmarks When Navigating without Vision

**DOI:** 10.1371/journal.pone.0072170

**Published:** 2013-09-05

**Authors:** Amy A. Kalia, Paul R. Schrater, Gordon E. Legge

**Affiliations:** 1 Department of Brain and Cognitive Science, Massachusetts Institute of Technology, Cambridge, Massachusetts, United States of America; 2 Department of Psychology, University of Minnesota Twin-Cities, Minneapolis, Minnesota, United States of America; Bielefeld University, Germany

## Abstract

This study investigated the interaction between remembered landmark and path integration strategies for estimating current location when walking in an environment without vision. We asked whether observers navigating without vision only rely on path integration information to judge their location, or whether remembered landmarks also influence judgments. Participants estimated their location in a hallway after viewing a target (remembered landmark cue) and then walking blindfolded to the same or a conflicting location (path integration cue). We found that participants averaged remembered landmark and path integration information when they judged that both sources provided congruent information about location, which resulted in more precise estimates compared to estimates made with only path integration. In conclusion, humans integrate remembered landmarks and path integration in a gated fashion, dependent on the congruency of the information. Humans can flexibly combine information about remembered landmarks with path integration cues while navigating without visual information.

## Introduction

As we travel in the world, we can use a number of features in the environment as landmarks to help determine our location. Yet these landmarks are not always visible, nor do we always pay attention to them as we travel. Imagine situations when we navigate without vision in the dark, while conversing with someone, or while looking at a mobile phone. To maintain a sense of where they are in such situations, humans rely on their estimates of the direction and velocity of travel obtained from vestibular, proprioceptive, and kinesthetic senses, here referred to as path integration. In these cases, do humans also use their memory of landmarks to navigate, or do they purely rely on path integration?

Landmarks are typically defined as visual objects in the environment that are salient, stable, and informative about location [1]–[3]. When landmarks are visible, humans can use a beaconing strategy of reducing distance to the landmark by directing their movements towards the goal location. Yet, it is often the case that landmarks are not visible from a starting location, in which case navigators need to rely on a remembered representation of the location of landmarks. Previous literature suggests that as humans become increasingly familiar with an environment, they can build a “cognitive map,” or a remembered representation of a space, that includes key locations and paths between locations [4]. Cognitive maps allow observers to navigate in the absence of directly perceived landmarks, as demonstrated in visually-directed walking tasks; participants can accurately view a landmark and then walk to it blindfolded, thereby using their memory of the landmark's location to guide their walk [5]–[7].

The current study explored the interaction between remembered landmarks encoded in a cognitive map and path integration. Previous studies with animals and humans suggest that visible landmarks are used when available, but path integration can be used as a backup reference system if landmark information is unreliable or not visible [8]–[12]. Observers can also keep track of their location relative to previously viewed landmarks while walking, as demonstrated by numerous spatial updating studies [13]–[16]. However, it is unknown if and how remembered landmarks influence the observer's perceived location.

Accordingly, we were interested in how observers use two cues to estimate their current location when navigating in a hallway environment without vision. We defined the cues as: 1) the remembered locations of landmarks based on the observer's cognitive map of the hallway and 2) the perceived distance and direction the observer walked from a starting location as determined by path integration. Observers obtained landmark information by briefly viewing a familiar hallway and a target location marked by a LED. They obtained path integration information by walking blindfolded to a location specified by the experimenter. We then asked observers to estimate their current location while still blindfolded. We also explicitly asked observers whether they believed they were at the target location viewed previously (i.e., whether the remembered landmark location matched the location specified by path integration).

On one hand, it is possible that observers only use path integration information when navigating without vision, suggesting that landmarks are only useful when visible. Another possibility is that observers use only one cue at a time; if observers believe they are near a remembered landmark after walking without vision, then their estimated location should be equivalent to the remembered landmark location. If they believe they have walked to a location that is different from the remembered landmark location, then their estimated location should be equivalent to the location specified by path integration. A third possibility is that observers integrate information from both sources of information. In a variety of perceptual tasks, humans integrate information from multiple cues as predicted by statistical models of cue combination [17]–[22]. The advantage of integrating remembered landmark and path integration information is that estimates may be more precise compared to only using path integration information. Philbeck and O'Leary [23] showed that remembered landmarks improve the precision (reduce the variability) of spatial localization estimates compared to having no landmarks in the environment. Interestingly, this effect occurs even though participants had no feedback about the remembered landmark's location as they approached it.

We also predicted that people integrate the location of a remembered landmark into their estimate only when their path integrator indicates that they are near it. In other words, when observers think they have walked to the remembered landmark, they integrate this information into their estimated location. In such a case, we will refer to path integration and remembered landmarks as providing congruent information about current location. When observers think they are not near the remembered landmark, they must continue relying on path integration to estimate location (remembered landmark location and path integration are incongruent). Such behavior would indicate that observers must continually keep track of their location relative to landmarks, but they only use this information to update their perceived location in a gated fashion. This outcome would suggest flexible integration of landmark and path integration strategies that tap into a common representation of space. Previous tests of cue integration models indicate that humans only integrate information from multiple cues if they provide congruent information. For example, humans will only integrate auditory and visual information about the spatial location of a target if both cues are perceived to come from the same spatial location [19]. If viewed landmarks are perceived to be incongruent with path integration, then animals and humans will only rely on path integration information to navigate [8], [11]

According to statistical models of cue combination, estimates based on the integration of information should be biased towards the more reliable cue. The noisier, or more variable, the estimates are from a particular cue, the less reliable that source of information is. Therefore, another aim of this study was to test whether integration of remembered landmarks and path integration is influenced by the quality of the visual landmark information. If remembered landmark information is susceptible to blur when viewing the target, we predicted that observers will be increasingly biased towards path integration information. However, if observers are relying on their previously acquired cognitive map to remember the landmark location, then the reliability of the visual information used to currently view the target may not affect judgments.

Importantly, our study differs from most cue combination studies because the cues were separated in time; participants had to retain landmark information in memory as they acquired path integration information. An earlier study by Brouwer and Knill [24] demonstrated that humans combine information acquired over time in a way that is consistent with statistical models of cue combination. In their study, observers combined visual and remembered information about a target's location during a reach as predicted by the reliabilities of each source of information. With this study as a precedent, our goal was to investigate how remembered landmark information and path integration are combined in a navigation task.

Previous studies testing cue integration in navigation have found that human adults average remembered landmark and path integration information, but the weights assigned to each cue are influenced by the order in which cues are presented [25]. Furthermore, the ability to integrate landmark and path integration information develops with age [26]. However, these previous studies did not investigate the effect of perceived cue congruency or cue reliability. As in other types of perceptual judgments, we expect cue congruency and reliability to influence integration of landmark and path integration cues.

**Figure 1:**
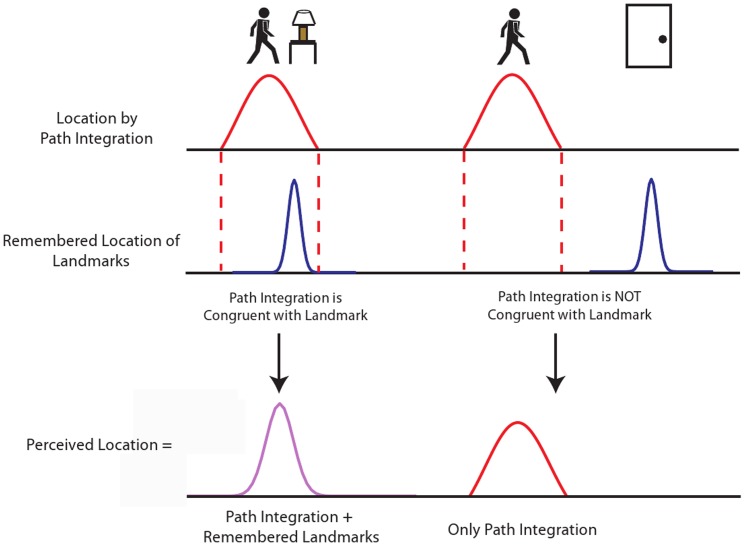
Predictions for integration of remembered landmarks and path integration. We predicted that when path integration and remembered landmarks provide congruent information about the observer's location, then these sources of information are combined. In this case, the estimate of location is more precise and biased towards the more reliable source of information. If remembered landmarks are incongruent with path integration, then only path integration is used to estimate location.

To summarize, this study tested whether humans combine remembered landmark and path integration information to localize themselves in an environment when visual information is unavailable, or whether they rely only on path integration. Specifically, we predicted that: 1) remembered landmarks and path integration information are only combined when they are perceived to be congruent, 2) unreliable (blurry) visual information alters how remembered landmarks are integrated into estimates of location, and 3) when remembered landmark and path integration information are combined, estimates of current hallway location are more precise than estimates based only on path integration information (Figure 1).

## Methods

### Ethics Statement

Our protocol was approved by the Institutional Review Board at the University of Minnesota Twin-Cities. All participants provided informed written consent.

### Participants

Nineteen normally-sighted observers (mean age = 21, 11 females/8 males) participated in this study. Participants were compensated monetarily or with extra credit in their psychology course.

### Materials

**Figure 2:**
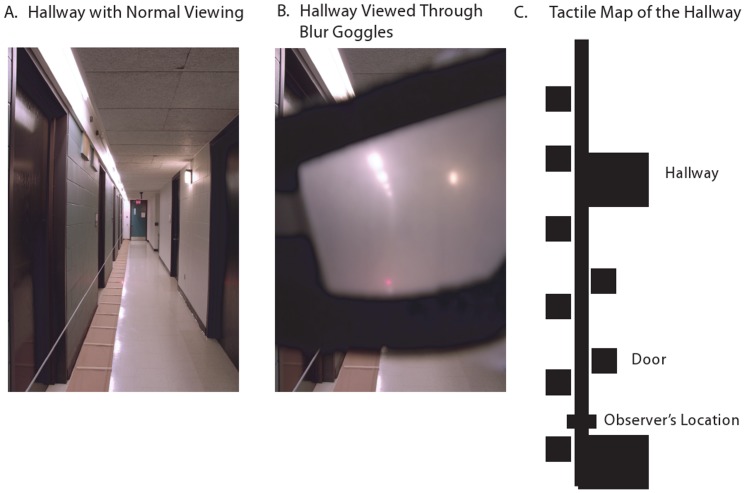
Test hallway and materials. Images of the hallway with (A) normal viewing, (B) viewed through the blur goggles, and (C) as represented by the tactile map.

Participants were tested in a building hallway approximately 15 meters in length under full lighting (Figure 2A). We marked target locations with single red, high-intensity Light Emitting Diodes (LEDs) embedded in wooden sticks that were placed on the floor every half meter down the length of the hallway.

We manipulated the reliability of viewed landmark information by having participants wear either clear or blurry monocular goggles. The blur goggles were made using Bangerter Occlusion foils [27] placed on the surface of the goggles. The blur foils produced an average logMAR acuity of 1.60 (Snellen acuity of approximately 20/800) and log contrast sensitivity of 0.23. The dominant eye was used during the experiment as determined by the Miles test (localization of an object with both eyes then one eye at a time [28]), while the fellow eye was occluded. When viewing the hallway through the blur goggles, it was not possible to see the textures on the floor and wall, nor the doors that were farther down the hallway (Figure 2B). The clear goggles were the same as the blur goggles, but without the blur foils.

The experiment required subjects to walk through the hallway while blindfolded. To prevent veering during these trials, participants held onto a steel cable that was strung along the length of the hallway. The cable was tight enough to prevent any slack from being a distance cue.

During walking trials, a laser range finder was used to measure the distance participants traveled. The laser range finder was connected to a laptop via a Bluetooth connection. Measurements were fed into a Kalman filter program that produced an auditory cue indicating when participants should stop walking. The algorithm used the participant's average walking velocity, measured by the experimenter prior to the start of the experiment, along with continuous measurements of the participant's distance during a walk to estimate when the participant would reach the specified distance. By using the algorithm, each trial could be automated rather than relying on an experimenter to stop the participant at the specified distance. On average, participants were stopped within +/−23 centimeters of the desired distance. Participants wore a white cardboard on their back, which provided a reflective surface for the laser. They also wore noise-reducing headphones to prevent the use of extraneous auditory cues, but could hear instructions from the experimenter and laptop via radio.

In all sessions, participants made location estimates on a tactile map that depicted the doors and intersections in the test hallway (Figure 2C). We used a tactile map so that the response modality was independent of the stimulus modalities (vision and walking); a visual or walking response could induce participants to give a higher weight to cues from the same modality during integration due to the ease of matching information within modalities compared to across modalities.

### Procedure

The experiment was conducted in four sessions, each lasting about one and a half hours. The sessions were conducted in the following order: 1) training and remembered landmark estimation in both viewing conditions (normal and blurry vision), 2) path integration estimation in both viewing conditions, 3) combined cue estimation in one viewing condition, 4) combined cue estimation in the remaining viewing condition. Half of the participants performed the no blur condition first, and the other half performed the blur condition first for each task. The remembered landmark estimation and path integration tasks are described in Experiment S1.

#### Training

Participants first performed a set of tests to evaluate their visual ability with the clear and blurry goggles. Visual acuity was measured with both goggles using a Lighthouse Distance Acuity test. Contrast sensitivity was measured with the Pelli-Robson Contrast Sensitivity chart. Participants were also tested on their ability to see the LED targets with the blurry goggles. Forced-choice testing confirmed that all subjects could detect the LED targets under all conditions with at least 90% accuracy. Participants were also trained on the hallway layout and the tactile map with normal vision prior to the blur conditions. Therefore, participants had some familiarity with the hallway prior to testing.

Participants also practiced walking blindfolded while holding on to the cable until they felt comfortable and were able to walk at a normal pace.

#### Combined Cue Estimation

Participants performed a cue conflict task with both remembered landmark and path integration information. For each single trial, participants stood at one end of the hallway and viewed a single LED target for as long as they needed to obtain a good idea of its location (typically less than 5 seconds). The target was viewed either through the clear or blurry goggles. The visual targets were located at 7 and 9 meters for twelve subjects, and at 5 and 11 meters for the other subjects (five subjects were tested at all four distances in separate sessions). Because of time restrictions, it was not feasible to test all the subjects on all four distances.

After viewing the target, participants pulled a blindfold over the goggles, and walked until they were stopped by an auditory cue. They then indicated their perceived location on the tactile map. Participants walked to locations that were the same as the visual target (conflict of 0 m) or differed by +/−0.25, 0.5, or 0.75 meters. They were told that they would be stopped either at the target or somewhere near it.

For each trial, participants also reported whether they thought the location they walked to was the same or different from the target location. Participants performed five trials for each visual target and conflict for a total of 70 trials in randomized order for each viewing condition.

### Data Analysis

We used robust linear models (with a bisquare estimator, implemented in the R statistical computing software) to obtain the best fits of the data. To determine whether information congruency and reliability shifted the reliance on remembered landmarks versus path integration, we used participants' estimates of their location in the hallway to measure the weight given to path integration. The weight of path integration information was equivalent to the slope of the line fitted to the participants' estimates of their walked location versus their actual walked location. For example, if participants only relied on path integration to judge their location (weight of path integration information is 1), then their responses should equal the walked distance (slope of 1). However, if participants believe they walked to the remembered landmark, and only relied on their memory of the landmark's location to estimate their current location (weight of path integration information is 0), then their responses should equal the distances of the visual targets; responses should not change with the walked distance (slope of 0).

We computed the weights of path integration information at each visual target distance (5, 7, 9 and 11 meters) by fitting separate lines to the data from the congruent vs. incongruent trials and the normal vs. blurry viewing conditions, and computed 95% confidence intervals on these slope coefficients. To test whether the weighting of path integration information was influenced by the reliability of remembered landmarks, we performed contrasts between the slopes of the fitted lines in the two viewing conditions (e.g. 7 m with normal viewing versus 7 m with blurry viewing). These analyses were conducted on the data grouped across participants.

For trials in which participants perceived remembered landmark and path integration to be incongruent, we predicted that the weight given to path integration information would be near 1. For trials in which participants perceived remembered landmark and path integration information to be congruent, we predicted an increase in the weight given to path integration information in the blurry viewing condition compared to the normal viewing condition.

We also evaluated the effect of information congruency and reliability on the precision of estimates. We measured the variability (precision) of estimates by computing the root mean square error of the residuals obtained from fits to individual participant estimates. We then computed the root mean square error for each participant in each viewing condition and for each target distance.

## Results

### Effect of Information Congruency and Reliability on Bias

**Figure 3:**
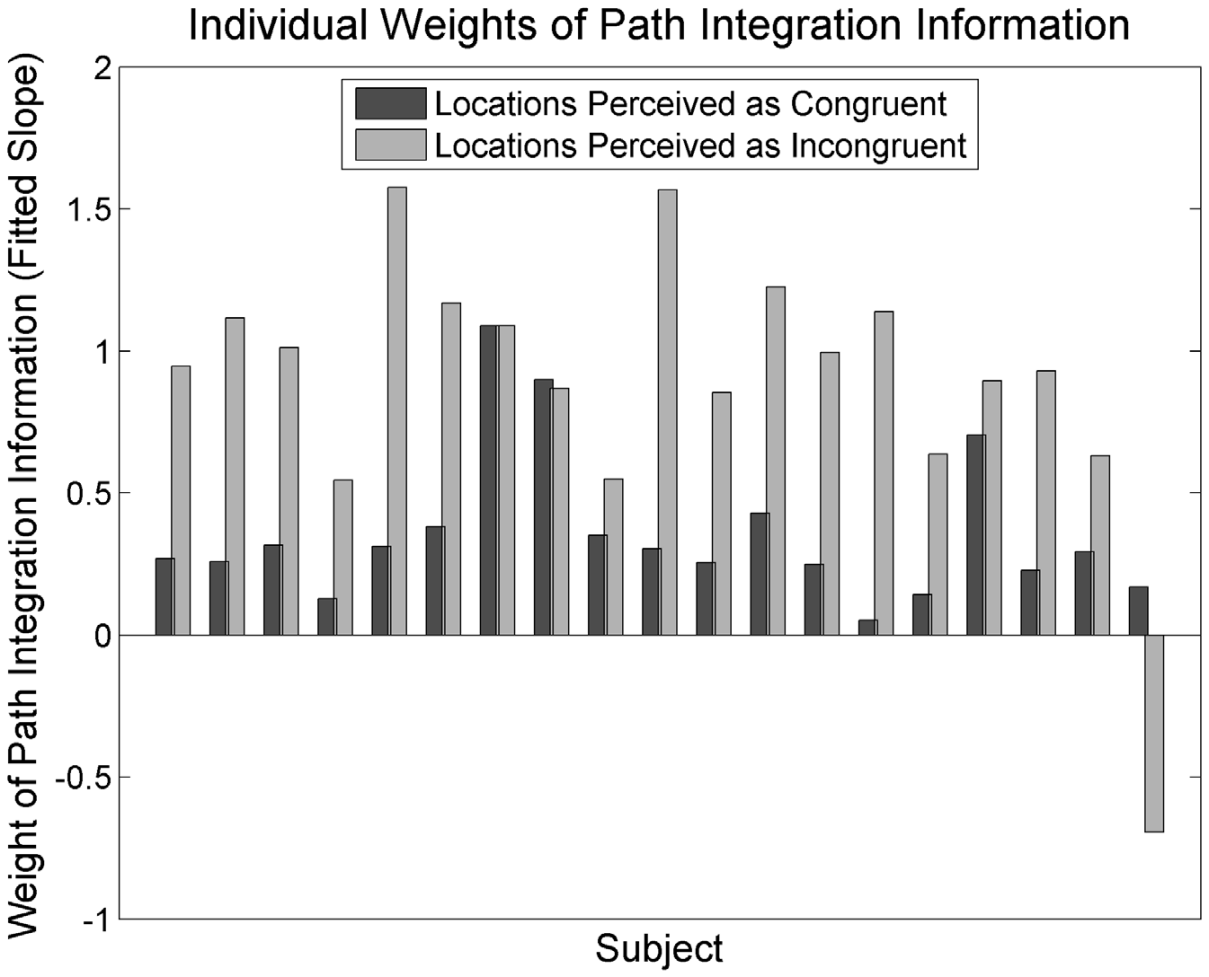
Participants' weighting of path integration in the combined cue task. Amount of weight (and 95% confidence interval) that participants gave to path integration in the combined cue task. Weights were computed from participants' estimates of their location in the hallway.

Figure 3 displays the weights of path integration information in the combined cue task, when both remembered landmark and path integration information were available. The weights were computed from participants' grouped estimates of their location in the hallway. When participants perceived remembered landmarks to be incongruent, they only used path integration to determine their location (weight of approximately 1.0). However, when they perceived remembered landmarks and path integration to be congruent, they integrated both sources of information in their estimates. Weights were approximately equal to 0.5 indicating that subjects averaged the information from remembered landmarks and path integration. Participants judged more trials to be incongruent as the discrepancy between the viewed and walked distances increased. For discrepancies between 0 and 0.25 meters, participants judged 38% of trials to be incongruent. For discrepancies between 0.5 and 0.75 meters, participants judged 48% of trials to be incongruent.

Except for the 9 meter target, viewing targets with blur did not alter how participants weighed remembered landmark versus path integration information. This result is surprising considering that blurry vision greatly impaired the precision of estimates made with only remembered landmarks (Figure S1 and Table S1). Together, these results suggest that remembered landmarks can bias perceived location when they are thought to be congruent with path integration information. However this bias is not affected by the reliability of remembered landmark information.

**Figure 4:**
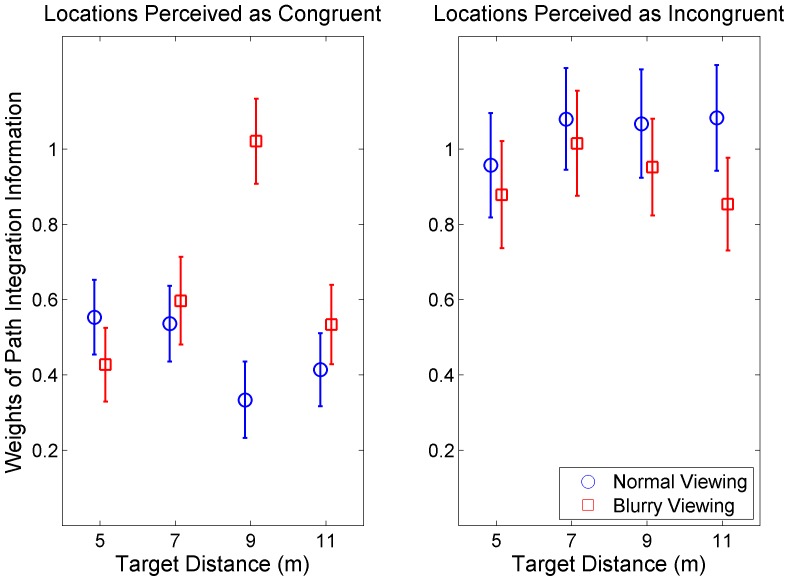
Weight given to path integration by individual participants. Amount of weight that individual subjects gave to location information obtained by path integration when viewed and walked locations were perceived as congruent versus incongruent.

The effect of perceived congruency is also apparent by looking at data from individual participants. Figure 4 illustrates individual weights of path integration information in trials perceived as congruent versus incongruent (estimates were averaged across viewing condition and target distance). The majority of participants show decreased weighting of path integration information when remembered landmark and walked locations were perceived to be congruent compared to when they were perceived to be incongruent.

### Effect of Information Integration on Precision

**Figure 5:**
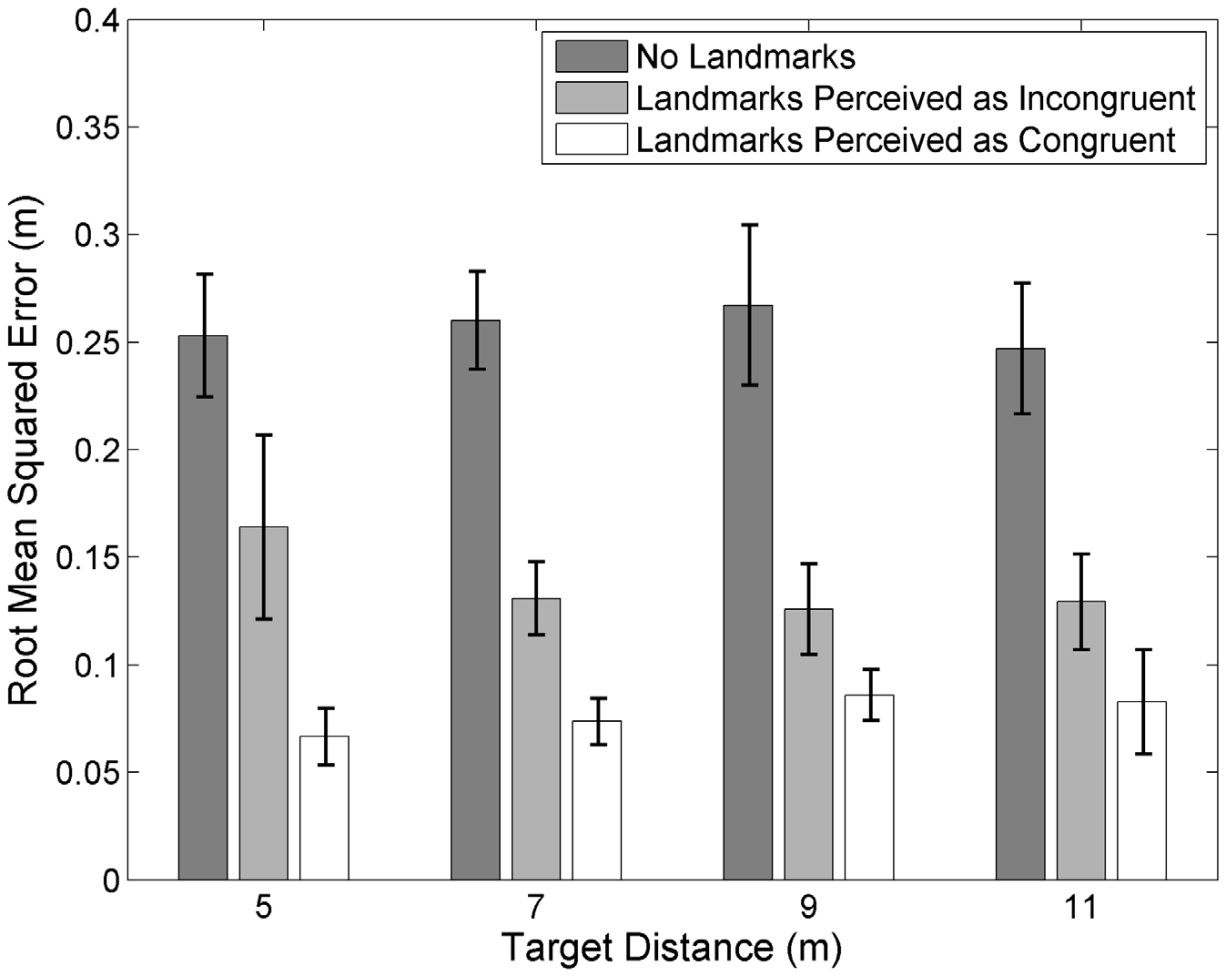
Precision of estimates in the combined cue task. The variability (root mean squared error) of localization estimates collapsed across viewing conditions when: 1) no landmarks, 2) remembered landmarks were judged to be incongruent with path integration information, and 3) remembered landmarks were judged to be congruent with path integration.

We conducted repeated measures analyses of variance on the root mean squares of participants' estimates, collapsed across blur levels since viewing targets with blur did not change the precision of estimates in a consistent way. We tested two within-subjects factors, remembered landmark condition (no landmarks, landmarks perceived as incongruent, and landmarks perceived as congruent) and target distance (7 and 9 meters or 5 and 11 meters). As predicted, we found that use of remembered landmarks increased the precision (decreased the variability) of localization estimates compared to when only path integration information was available (F(2,24) = 49.59, p<0.001 for 7 and 9 m targets, F(2,22) = 21.26, p<0.001 for 5 and 11 m targets, Figure 5). Interestingly, precision increased even when remembered landmarks were judged to be incongruent, although not to the same extent as congruent landmarks.

## Discussion

This study investigated whether humans combine information from remembered landmarks and path integration to estimate their current location in a real-world environment when navigating without vision. Our results show that observers do integrate information from remembered landmarks and path integration in some situations. We find that integration is dependent on the congruency, but not the reliability, of the information. When participants perceived the remembered landmark location to be different from the walked location, they only relied on path integration information to estimate their location. Yet when the remembered landmark and walked locations were perceived to be congruent, participants integrated remembered landmark information into their estimates as seen by the decreased reliance on path integration information. Interestingly, incongruent remembered landmarks still improved the precision (reduced uncertainty) of location estimates compared to path integration alone, even if they did not bias these estimates. However, congruent remembered landmarks reduced uncertainty even more.

One possibility why incongruent remembered landmarks reduced uncertainty is that observers still updated their position relative to their memory of the landmark location (e.g. “I have walked past landmark A but am not yet at landmark B”). Therefore, the remembered landmark information was still informative about position. Alternatively, perhaps observers give partial weight to remembered landmark information depending on congruency. In other words, the weighting given to a remembered landmark may be a function of how near it is perceived to be.

The exploration of cue congruency has important theoretical implications for understanding how the perceptual system integrates information from multiple sources. Studies exploring causal inference in sensory integration suggest that the perceptual system should treat noticeably discrepant cues as coming from different sources, and therefore this information should not be integrated [19]. Although the underlying computation is the same (separation versus integration of information), determining the congruence of information is a different problem. In this study, remembered landmark and path integration information clearly came from two different sources that were separated temporally. The question of interest was whether both sources provided information that was relevant to localization. We measured relevance as the spatial congruence of the position estimates provided by remembered landmarks and path integration.

Our demonstration of gated integration of remembered landmarks and path integration suggests that these strategies are strongly intertwined. When they believe they have reached a landmark, observers update their perceived position by combining the remembered landmark location with their perceived walked location. This behavior indicates that when observers walk without vision, or when they are not paying attention to their visual surroundings, they continuously keep track of their position with respect to remembered landmarks in the environment. Our results complement findings that altering the mappings between visual motion cues and path integration effects subsequent judgments of self-motion without vision, suggesting that a single multimodal representation of space underlies large-scale navigation [29].

We also found that usually the reliability of the cues did not influence how much they were weighed in the final judgment. Regardless of the quality of visual information, observers averaged the location estimates provided by path integration and congruent remembered landmarks as in previous studies that did not manipulate visual reliability [25]. One exception was that observers weighed path integration more when the 9 meter target was viewed with blurry versus normal vision. There is no clear explanation why the 9 meter target would have been treated differently than the other distances.

The effect of blurred viewing of landmark information may depend on the strategy participants used to estimate the location of the target. We can consider two possible strategies. In Strategy A, participants viewed the target and used the current percept to estimate the location of the target relative to themselves and other features in their cognitive map. In this case, their memory of the target's location was based on the perceptual information available on a trial-by-trial basis, which should be less reliable with blurry vision. In Strategy B, participants previously learned the locations of the targets and incorporated them into their cognitive map. When participants viewed the target during a particular trial, they made a categorical judgment about which target they were looking at and then relied on their cognitive map to remember the location of the target. In this case, memory of the target's location was not based on the current percept and therefore the information was equally reliable with or without blurry vision. Since we did not see an effect of blur on participants' localization estimates in the combined cue task, it may be that they were performing the task as described in Strategy B. They may have quickly learned the locations of the targets during the course of the experiment, or during measurements of single-cue reliability (see Experiment S1). Other possible reasons why blurry vision did not affect behavior are also discussed in Experiment S1 in the Supporting Information section.

Regardless of these other potential factors, it is still remarkable that blurry vision did not alter how participants combined information from remembered landmarks and path integration when estimating their location. This finding agrees with previous work demonstrating equivalent performance in spatial updating when landmarks are viewed with or without blur [30]. The robustness of navigation behavior to degraded vision is consonant with the ubiquity of accurate spatial updating across phyla with markedly different acuity [31], but it remains an interesting puzzle that needs to be investigated further.

Manipulating the visual factors of acuity and contrast also has clear implications for understanding how individuals with low vision combine sensory information to estimate their location. People with visual impairment do not express increased sensitivity to non-visual information obtained by walking, as demonstrated by studies of path integration with blind individual [32]. Yet, it may be that with experience people with low vision adjust how they weigh visual and non-visual cues to obtain the most optimal perceptual estimates given the available information. Exploring whether people with visual impairment optimally integrate residual vision with other sensory information can be a useful test of the effectiveness of mobility training.

Although this experiment tested the integration of remembered landmarks when walking without vision, we believe that our findings do apply to navigation with vision but when landmarks are not visible from the observer's current location (for instance, due to occlusion or distance). The main difference between walking with eyes open versus closed is likely the precision with which observers perceive the nearness of the landmark location relative to their current position. Once the landmark is viewable, a beaconing strategy can be used to approach it, but until then observers must maintain a sense of their location relative to the landmark using path integration. It seems that the value of a landmark in spatial updating depends on both the precision of our remembered knowledge of its location in the layout and also the precision with which we perceive the landmark relative to our current position.

In conclusion, this study provides evidence that humans do integrate information from remembered visual landmarks and path integration to determine their location in an environment. Integration is dependent on the congruence of the information- humans will only incorporate remembered landmarks that fall within the range of locations specified by path integration. However, even incongruent remembered landmarks reduce uncertainty about location. Furthermore, integration is not dependent on the reliability of remembered landmark information. Instead, use of remembered landmark information for localization is remarkably robust to blurred vision.

## Supporting Information

Experiment S1 **Reliability of Remembered Landmark and Path Integration Information.**
(DOC)

Figure S1 **Participants' estimates in the single cue tasks.** Remembered landmark and path integration estimates in the normal and blurry viewing conditions compiled across participants. Data points marked with an ‘x’ were considered to be outliers according to the robust fits.
(TIF)

Figure S2 **Remembered landmark estimates with and without a delayed response.** Remembered landmark estimates in the normal and blurry viewing conditions with and without a delay. Data is compiled across participants.
(TIF)

Table S1 **Variability of cues and predicted weights.** Variability (root mean square errors) and predicted weights of path integration information in the combined cue task computed from the remembered landmark and path integration estimation tasks for both viewing conditions at each of the target distances.
(DOC)
