# Supplementary material for: Combining Path Integration and Remembered Landmarks When Navigating without Vision
Source: PLoS One. 2013 Sep 5;8(9):e72170. doi: 10.1371/journal.pone.0072170 (PMC3764103; doi:10.1371/journal.pone.0072170)
Supplement: Experiment S1 — Reliability of Remembered Landmark and Path Integration Information. (DOC) [file pone.0072170.s001.doc]

**Experiment S1A**

We attempted to quantify the reliability of remembered landmark and path integration in the following single-cue tasks. This allowed us to determine the predicted weighting of each source of information according to statistical models of cue combination.

**Methods**

**Participants.** All the participants who were tested in the cue combination task described in the main article were also tested in the single-cue experiments.

**Apparatus.** The test environment and the other materials were the same as in the experiment described in the main article.

**Remembered Landmark Estimation*.*** This task was used to measure the reliability of the remembered landmark cue when targets were viewed with normal versus blurry vision. This task also confirmed that subjects could accurately use the tactile map to indicate the location of the targets.

Participants stood at one end of the hallway and viewed a target located 5, 7, 9 or 11 meters away. They were allowed to look at the target as long as they needed to obtain a good idea of its location. Then, they pulled a blindfold over the goggles, and indicated on the tactile map the location of the remembered target. Participants performed ten trials at each target distance, plus an additional six trials at alternate distances to prevent memorization, for a total of 46 trials in a randomized order for each viewing condition.

**Path Integration Estimation.** This task was used to measure the reliability of path integration for localization. Participants viewed the hallway through clear or blurry goggles, then wore the blindfold, and walked until stopped by an auditory cue. Next they indicated their perceived location on the tactile map while still wearing the blindfold. The purpose of the hallway view was to allow participants to refresh their visual memory (i.e. cognitive map) of the environment on each trial, thereby mimicking conditions in the combined cue estimation task. Participants performed 40 trials in each viewing condition, ten trials for each target distance (5, 7, 9, and 11 m) in a randomized order.

**Results**

Variability for single-cue estimates was measured by first fitting separate lines to the responses from each subject for each cue (remembered landmark and path integration) and viewing condition (normal and blurry). Variability (*σ_v_* and *σ_w_*) was calculated as the root mean square of the residuals of the individual best fit lines grouped across subjects. By using the residuals from fits to individual subject data to compute variability, we could minimize the influence of subject biases (tendency to overshoot or undershoot location estimates) from our estimates of response variability across subjects.

Figure S1 depicts responses for all participants in the remembered landmark and path integration estimation tasks. The variability of the estimates for each condition is displayed in Table S1. Remembered landmark estimates were more variable with blurry viewing compared to normal viewing. Viewing condition did not have as great an impact on path integration estimation. The variability of the single cue estimates (*σ_v_* and *σ_w_*) were used to predict the weights (*w_w_*) of these cues in the combined cue task. We computed the predicted weights based only on the data from subjects tested at corresponding distances in the combined cue task. The predicted weights, also shown in Table S1, indicate that path integration should have a greater weighting with blurry vision compared to normal vision when remembered landmarks and path integration information are integrated.

**Discussion**

In the experiment described in the main article, participants weighed path integration information more than predicted by the single-cue reliability measurements described here when they viewed targets with normal vision. One possible explanation for the increased reliance on path integration is that the reliability of remembered landmarks decreases over time due to memory decay. Accordingly, our estimates of remembered landmark reliability as measured in the single cue visual task would inaccurately represent this information as being more reliable than it actually is. We tested this possibility in Experiment S1B in which participants made remembered landmark estimates after a time delay.

**Experiment S1B**

The cue combination task required participants to learn a cognitive map of the environment and to maintain the locations of visual landmarks in memory while walking. How well is the remembered location of the visual landmark preserved during the walk? Previous research on visual memory decay suggests that either visual memory accumulates noise over time [1] or decays in a more deterministic fashion [2]. The goal of this experiment was to investigate if the reliability of remembered landmarks for localization decays over time.

**Method**

**Participants.** We tested six normally-sighted participants (mean age = 22, 2 females/4 males) who were compensated monetarily or with extra credit in their psychology course.

**Apparatus.** The test environment and the other materials were the same as in the experiment described in the main article.

**Procedure.** Participants performed the remembered landmark estimation task, described in Experiment 1, in four conditions: two viewing conditions (normal versus blurry viewing) and two delay conditions (no delay versus delay). The order of the conditions was randomized for each participant. The no delay condition was the same as the remembered landmark task described above. In the delay condition, participants viewed the target, wore the blindfold, and waited until they heard an auditory cue before responding on the tactile map. The delay corresponded to the amount of time the participant needed to walk to the target (target distance (m) / walking velocity (m/s)). We measured each participant’s walking velocity (mean = 1.03 m/s) to calculate these response delays. Time delays on average ranged from 4.85 seconds for targets at 5 meters to 10.68 seconds for targets at 11 meters. Participants performed 46 trials in each condition, 10 trials at 5, 7, 9, and 11 meters and 6 trials at alternate distances to prevent memorization.

**Data Analysis.** Robust linear models were fit to each participant’s data in each of the four conditions (no delay versus delay, normal and blurry viewing). We computed the variability of estimates as the root mean of the weighted squared residuals across all target distances. To test the effect of delay, we performed paired t-tests comparing the variability of estimates between the no delay and delay conditions for each viewing condition.

**Results**

The variability of estimates in the four conditions, averaged across participants and target distances, were as follows: normal viewing - no delay: 0.023 m, normal viewing - delay: 0.022 m, blurry viewing – no delay: 0.093 m, blurry viewing – delay: 0.109 m (Figure S2). Paired t-tests revealed no significant differences between the no delay and delay conditions in both viewing conditions.

**Discussion**

This experiment revealed that the reliability of remembered landmark information for target locations did not decrease over the time needed to walk the same distance. We can conclude that the delay between viewing the target and responding in the combined cue task of described in the main article does not account for the greater than predicted reliance on path integration information. Although longer delays or distractor tasks could be more detrimental to the visual memory of targets, previous studies show that memory for landmarks is highly robust over time, and remains accurate even a year after learning [3]. The results of this experiment corroborate these previous findings.

There are still three potential factors that we did not account for when measuring the reliability of remembered landmark and path integration information that may have altered our predictions. First, the reliability of remembered landmarks may decrease over time due to memory decay. We found in this experiment that the reliability of remembered landmark information remained consistent during short time intervals. Previous studies have also found that remembered landmark information remains robust over time, more so than path integration [3, 4]. Perhaps encoding landmarks into a cognitive map allows this information to be stable over significant time delays.

Second, the order in which landmark and walked locations were presented may have altered their reliabilities. Unlike typical cue conflict studies, participants in our experiment were presented with information sequentially- they first viewed a target and then walked to it. Ellard and Shaughnessy [5] found that the order of presentation influenced how visual and walking information were combined to estimate distances. When the walking information was presented last, participants weighed both cues about equally. When the visual landmark information was presented last, the visual weighting was significantly greater than walking information. Therefore, it is possible that the order of information influenced how participants weighed walking information in our experiment.

Third, subjects possibly translate their visual estimate of the target location into a motor plan, and therefore the variability of the visual estimate could only be as precise as walking estimates of location. Therefore, reliability of visual estimates with normal vision would have been overestimated in our single-cue task. However, past work suggests that the variability of walking estimates of a target’s location does increase when the target is viewed with a similar level of blur as the current experiment [6, 7]. This suggests that even if our measurements of visual reliability were overestimated, there would still be a difference in the variability of walking estimates implemented by the motor plan when the target was viewed with or without blur. Therefore, this explanation cannot fully account for the lack of a change in weightings with blur in the combined task.

**References**

1. Kinchla RA, Smyzer F (1967) A diffusion model of perceptual memory. Percept Psychophys 2(6):219-29.

2. Gold JM, Murray RF, Sekuler AB, Bennett PJ, Sekuler R (2005) Visual memory decay is deterministic. Psychol Sci 16(10):769-74.

3. Stankiewicz BJ, Kalia AA (2007) Acquisition of structural versus object landmark knowledge. J Exp Psychol Human 33(2): 378-390.

4. Ziegler PE, Wehner R (1997) Time-courses of memory decay in vector-based and landmark-based systems of navigation in desert ants, Cataglyphis fortis. J Comp Physiol A 181:13-20.

5. Ellard CG, Shaughnessy SC (2003) A comparison of visual and nonvisual sensory inputs to walked distance in a blind-walking task. Perception 32(5):567-578.

6. Tarampi MR, Creem-Regehr SH, Thompson WB (2010) Intact Spatial Updating with Severely Degraded Vision. Atten Percept Psycho 72(1):23-27.

7. Kalia AA, Schrater PR, Legge GE, Kallie CS (2008) Estimating absolute distances with blurred vision. J Vision 8(6):1047.
