# Supplementary figures and images for: Combining Path Integration and Remembered Landmarks When Navigating without Vision
Source: PLoS One. 2013 Sep 5;8(9):e72170. doi: 10.1371/journal.pone.0072170 (PMC3764103; doi:10.1371/journal.pone.0072170)

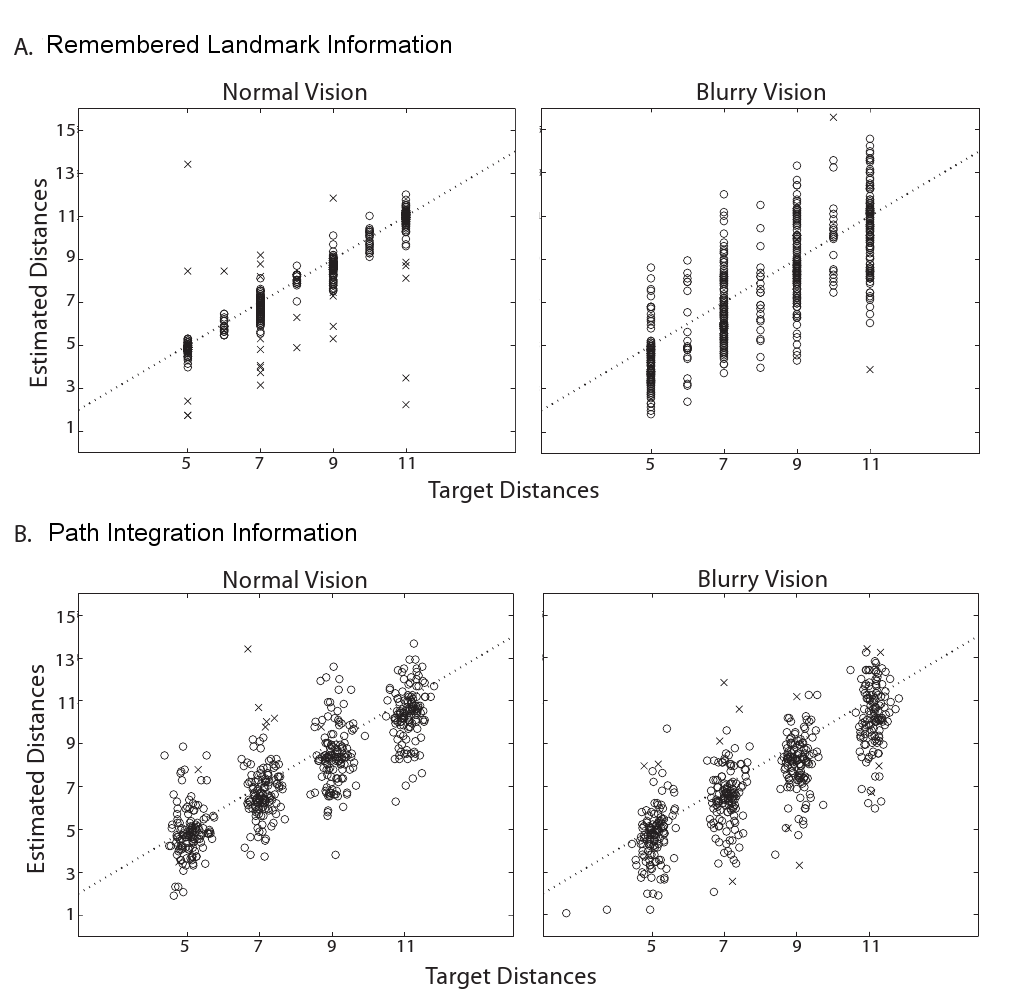

Supplement: Figure S1 — Participants' estimates in the single cue tasks. Remembered landmark and path integration estimates in the normal and blurry viewing conditions compiled across participants. Data points marked with an ‘x’ were considered to be outliers according to the robust fits. (TIF) [file pone.0072170.s002.tif]

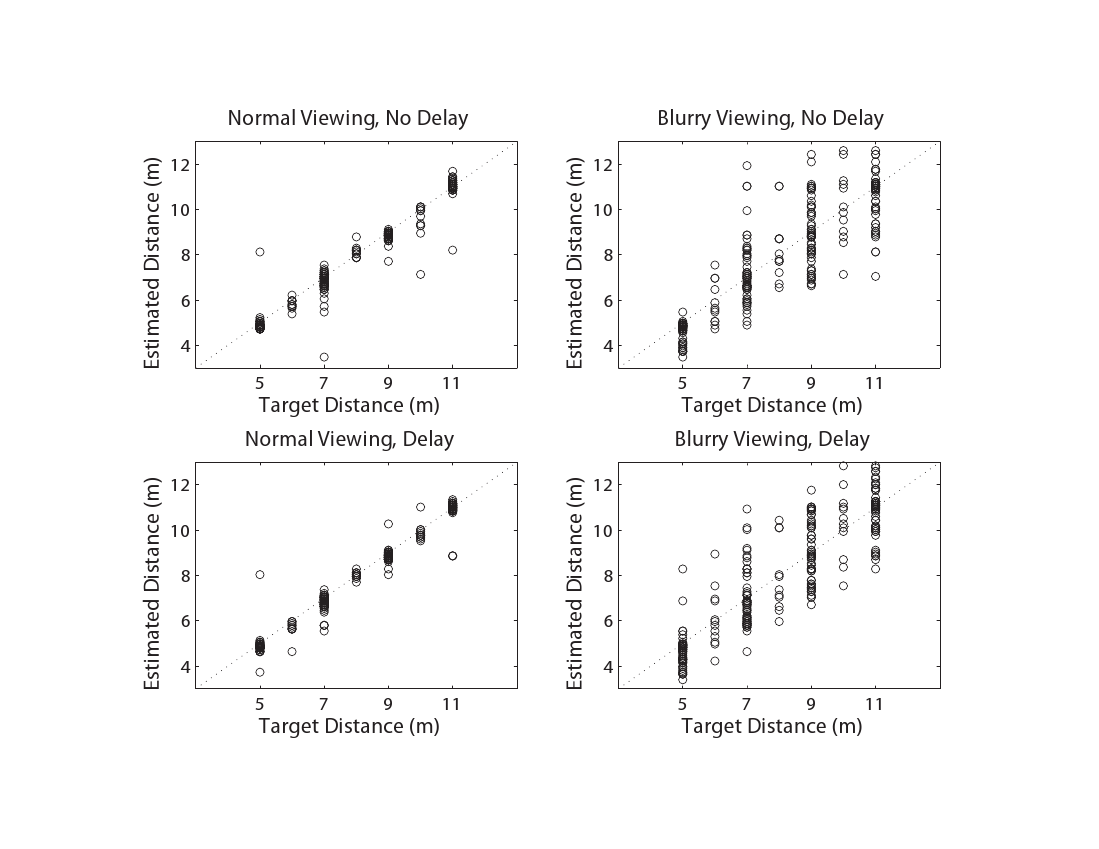

Supplement: Figure S2 — Remembered landmark estimates with and without a delayed response. Remembered landmark estimates in the normal and blurry viewing conditions with and without a delay. Data is compiled across participants. (TIF) [file pone.0072170.s003.tif]
