# Supplementary material for: Combining Path Integration and Remembered Landmarks When Navigating without Vision
Source: PLoS One. 2013 Sep 5;8(9):e72170. doi: 10.1371/journal.pone.0072170 (PMC3764103; doi:10.1371/journal.pone.0072170)
Supplement: Table S1 — Variability of cues and predicted weights. Variability (root mean square errors) and predicted weights of path integration information in the combined cue task computed from the remembered landmark and path integration estimation tasks for both viewing conditions at each of the target distances. (DOC) [file pone.0072170.s004.doc]

**Table S1.**

|  | Variability of  Remembered Landmark Estimation  (*σ_v_*) | Variability of  Path Integration Estimation  (*σ_w_*) | Predicted Weights of  Path Integration Information $w_{w}=\frac{1/\sigma_{w}^{2}}{1/\sigma_{v}^{2}+1/\sigma_{w}^{2}}$ |
| --- | --- | --- | --- |
| Normal Viewing | 5 m: 0.030 m  7 m: 0.030 m  9 m: 0.026 m  11 m: 0.043 m | 5 m: 0.091 m  7 m: 0.068 m  9 m: 0.084 m  11 m: 0.079 m | 5 m: 0.10  7 m: 0.16  9 m: 0.09  11 m: 0.23 |
| Blurry Viewing | 5 m: 0.074 m  7 m: 0.079 m  9 m: 0.090 m  11 m: 0.101 m | 5 m: 0.082 m  7 m: 0.081 m  9 m: 0.074 m  11 m: 0.070 m | 5 m: 0.45  7 m: 0.48  9 m: 0.60  11 m: 0.67 |
